# British Hospitals and Paying Patients

**Published:** 1907-01-26

**Authors:** 


### HOSPITAL ADMINISTRATION.

### CONSTRUCTION AND ECONOMICS.

### BRITISH HOSPITALS AND PAYING PATIENTS.

#### PAY PAVILIONS AND AN INSURANCE POLICY.

It may be thought remarkable that the English nation has not yet met the urgent need which exists for hospital treatment for those who wish and can afford to pay a moderate sum for such accommodation. It may be well to state at the outset, therefore, that the true explanation is to be found in the financial difficulties which present themselves in connection with this problem.

For 30 years past, at least, anyone could acquire a measure of popularity by advocating the provision of hospitals for paying patients at a cost of from one guinea a week upwards. The impracticability of such proposals becomes apparent when tested by the average cost of treating a patient in the wards of a great general hospital in London, which, at a moderate computation, is about two guineas per week. The late Mr. John Walter was one of the very first advocates of the pay system, and he was one of the original promoters of the Home Hospitals Association, which opened the first Pay Hospital, at Fitzroy House, Fitzroy Square, in 1880. This hospital was rebuilt about three years ago, and now contains 30 beds. It has been self-supporting from the outset. The experience of Fitzroy House shows that the minimum cost of providing adequate accommodation " in a healthy and quiet neighbourhood, not too far from the locality in which public opinion condemns the majority of surgeons to reside," cannot, as a business proposition, be less than four guineas a week, irrespective of the doctor's fees. In practice it has been found that the large majority of patients, who desire, and ought undoubtedly to have, hospital accommodation provided for them on a paying basis, cannot afford as much as four guineas a week. It follows that if one large pay hospital were to be built, on the conditions proposed, it must, to succeed financially, admit for the most part patients of a higher social status than the classes we have indicated; or it must prove in practice a failure.

On the Continent the difficulties here indicated have been overcome by a system which grades the patients, and provides that each class shall pay something, though not necessarily a remunerative rate. Thus, the cheapest accommodation is available for the poor, who are paid for by the commune or the municipality. The next rate is rather higher, though not necessarily a remunerative one; then comes a rate which is inclusive, and represents the actual cost of the treatment, or rather more; and the highest grade of all yields a profit on the fees which are charged. Seeing that such hospital systems are supported mainly by the Government, the municipalities, or the local authorities, the financial problem becomes simple, and the system has the further advantage, that no one need forfeit his self-respect by accepting free hospital treatment. In the United States most of the large hospitals have pay wings or pavilions, containing private wards, and the room rates per week, including board and divided attention of nurse, and ordinary dressings and medicines, range from £15 per week (with a private bathroom) to £5 per week. The accommodation at the lower rate is very limited, for in a large institution like the Roosevelt Hospital, which contains 46 private wards, there are only two where the rates are as low as £5 per week. In the Colonies a similar system prevails. Some American hospitals are supported entirely by the municipality, for instance the Boston City Hospital, which is one of the best organised and administered. At this latter hospital the income received from paying patients, according to the last returns (1904), was rather under £14,000, of which £5,000 was paid for the board of patients by the State of Massachusetts, and £1,300 by various cities and towns, for patients these authorities sent to the City Hospital, showing that the continental system is partially followed in America. On the Continent, in the United States, and in the Colonies, there are a large number of very efficient pay hospitals run by doctors or private individuals, and here the rates charged are frequently higher than those quoted at the Roosevelt Hospital.

It will be seen that the needs of the middle and lower middle classes in the countries named are met by hospital provision in separate wings erected on a portion of the site under the jurisdiction of hospitals which are supported as voluntary or municipal or State institutions. Years ago there was a movement in favour of establishing pay wings in connection with the larger general hospitals in this country, and the London Hospital at Whitechapel took power in a special Act, which received the Royal assent on May 19th, 1884, to admit paying patients to its wards, to an extent not exceeding 10 per cent. of the total beds. The movement did not become general, and little progress in this direction has been made. Yet the practical solution of this problem can only be brought about by erecting private ward pavilions in juxtaposition to some of the large general hospitals, the entrances to which shall be quite distinct from that for free patients, although the pay wards would be managed and worked by the general hospital staff. If this were to be done, exhaustive and careful estimates go to prove that patients might receive adequate accommodation and treatment, including medical attendance, for a fee of from three guineas a week. In order to provide such medical attendance three things are essential. A proportion of all the fees received must be pooled, or insurance arrangements made, to provide the doctors' fees; each patient must have the right to select his own medical attendant; and the fees of the doctors must be on a reduced scale, fixed on some such basis as that whereby a number of consulting practitioners in Birmingham have agreed to attend artisans and others at reduced fees on certain prescribed conditions.

Under any system, however, it is probable that many patients could not afford to pay even reduced fees for medical attendance. To overcome this difficulty, actuarial rates have been prepared, by which anyone who chooses to be provident, can insure for a reasonable premium, so as to cover hospital treatment and medical attendance during each year, if and when required. This scheme has taken a great deal of time and thought, but it is now so far advanced as to prove that the premiums payable for a hospital policy, including the operation fees, can be as reasonable as those charged by accident and other insurance offices, being thus well within the means of the middle and lower middle classes.

It is fair to conclude, in these circumstances, that the solution of this problem has at length assumed a practical shape in every respect save one. It is of course impossible to reduce any such scheme to practice, unless arrangements can be made to provide private ward pavilions in connection with those general hospitals which are willing and able to afford facilities for their erection and management. It has been found that some of the general hospitals would be willing to provide a site where possible, and to undertake the management of the private pavilions for pay patients, providing the cost of the erection and equipment of such pavilions is forthcoming. It is calculated that the minimum fee of three guineas per patient per week would provide a sum for interest equal to 4 per cent. on the cost of the buildings and their equipment, 1 per cent. of which might constitute a sinking fund for the repayment of the capital sum. If £250,000 were available for the erection of private wards on the conditions mentioned, a thorough system of pay hospitals could be brought into practical working within the next three years.

The hospitals which agreed to have pay pavilions would possess the economic advantage of being able to provide that the circumstances of every patient admitted to a free bed should be carefully inquired into, and, when found able to pay, such patients could be removed to the paying beds. It is not improbable that a few wealthy men may be disposed, like the late Mr. Peabody, to co-operate in a scheme of this kind by advancing the money to erect the first private pavilions. If so the scheme should soon prove self-supporting and self-extending. If once this system were established, the need for hospital accommodation felt by every class of the community in this country could be adequately met, and under it, the numerous existing abuses and evils attached to the present system of hospital relief would disappear.

